# Spontaneous low-protein intake in older CKD patients: one diet may not fit all

**DOI:** 10.3389/fnut.2024.1328939

**Published:** 2024-02-14

**Authors:** Simone Vettoretti, Paolo Molinari, Silvia Armelloni, Giuseppe Castellano, Lara Caldiroli

**Affiliations:** ^1^Unit of Nephrology, Dialysis and Renal Transplantation - Fondazione IRCCS Ca’Granda Ospedale Maggiore Policlinico di Milano, Milan, Italy; ^2^Department of Clinical Sciences and Community Health, University of Milan, Milan, Italy

**Keywords:** protein intake, malnutrition, oral supplementation, chronic kidney disease (CKD), low protein diet

## Abstract

**Background:**

Protein restriction has been extended to stage 3 chronic kidney disease (CKD) regardless of age in the latest K-DOQI guidelines for the dietary management of patients with CKD. However, in elderly CKD patients there is a tendency to a spontaneous reduction in protein and energy intake that may impair the overall nutritional status. The aim of our study is to assess whether there are differences in malnutrition, exercise capacity and inflammatory status in elderly CKD patients with spontaneously low protein intake (sLPI) compared with patients with normal protein intake (NPI).

**Methods:**

We performed a cross-sectional analysis of 123 incident patients. Malnutrition was assessed using Malnutrition Inflammation Score (MIS) and serum markers; As for physical performance, we used Short Physical Performance Battery (SPPB) and handgrip strength.

**Results:**

We found that in older patients with advanced CKD, as many as 68% had low spontaneous protein intake, and they were more malnourished evaluated with MIS (25% vs. 10%, *p* = 0.033), protein-energy wasting (PEW) (43% vs. 14%, *p* = 0.002) and nPCR (0.63[0.51–0.69] vs. 0.95[0.87–1.1], *p* < 0.0001). They also had worse body composition, in terms of lower mid-arm muscular circumference (MAMC), fat tissue index (FTI) and higher overhydration (OH). sLPI patients also had higher levels of IL6 (4.6[2.9–8.9] vs. 2.8[0.8–5.1], *p* = 0.002). Moreover, sLPI patients were frailer (33% vs. 24%, *p* = 0.037) and had poorer physical performance especially when assessed with (SPPB) (7[5–9] vs. 9[7–10], *p* = 0.004) and gait test time (6.08 + 2 vs. 7.22 + 2.7, *p* = 0.04). sLPI was associated with lower physical performance [SPPB OR, 0.79 (0.46–0.97), *p* = 0.046] and malnutrition [MIS 1.6 (1.05–3.5), *p* = 0.041] independently from patients’ age and eGFR.

**Conclusion:**

We found that in older patients with advanced CKD, up to 68% had low spontaneous protein intake and were frailer, more malnourished and with lower physical performance. These findings emphasize the importance of assessing patients’ needs, and personalized approaches with individual risk–benefit assessments should be sought. To achieve the best possible outcomes, targeted interventions should use all available tools.

## 1 Introduction

Latest K-DOQI guidelines on dietary management of patients with chronic kidney disease (CKD) extend the range of dietary interventions, particularly protein restriction, to stage 3 CKD regardless of age, and emphasize that optimization of protein intake is associated with reduced mortality and morbidity (1). However, in elderly CKD patients there is a tendency to a spontaneous reduction in protein and energy intake (2–5) that may impair the overall nutritional status. Therefore, the necessity to maintain a balance between the preservation of renal function and the prevention of malnutrition, may lead in clinics to therapeutic minimalism. Thus, nutritional interventions in elderly patients with CKD are far from being straight forward and homogeneously applicated (6).

The indications to prescribe a low protein diet irrespectively of age, as reported in the recent K-DOQI guidelines, are in apparent contradiction with the dietary indications for the elderly that have been expressed by other scientific societies. In particular, the recent ESPEN guidelines suggest that high-protein diets may help to counteract sarcopenia and malnutrition in the elderly (7, 8).

The aim of our study was to assess the prevalence of elderly patients with advanced CKD with a spontaneous reduction of protein intake among those that are incident in an outpatient CKD clinic. Furthermore, we evaluated whether in patients with spontaneously reduced protein intake (sLPI) there was any difference in malnutrition, sarcopenia and systemic inflammation when they were compared to patients with normal protein intake (NPI). These data will possibly help to develop tailored nutritional approaches and educational interventions based on individual risk–benefit assessment in elderly patients with advanced CKD.

## 2 Materials and methods

### 2.1 Patients and study design

Between 9/2016 and 3/2018, 123 incident elderly CKD patients attending our outpatient clinic were evaluated in this cross-sectional study. Inclusion criteria were: age ≥ 65 years, CKD stages 3a to 5 on conservative therapy and relatively stable eGFR over the previous 6 months (with less than 2 mL/min/1.73/m^2^ variation). eGFR was estimated using the CKD-EPI formula. To eliminate potential confounders, we excluded patients with cancer, cirrhosis and/or ascites. We also excluded patients taking immunosuppressive drugs and those with severe heart failure (NYHA class III-IV), nephrotic syndrome, thyroid disease, inflammatory bowel disease, and inability to cooperate. Patients who had been hospitalized in the previous 3 months were also excluded. Biochemical and urinary parameters were collected on the morning of the index visit after an overnight fast of at least 12 h. Written informed consent was obtained from the individuals and the study was conducted in accordance with the ICP Good Clinical Practice Guidelines. The study was approved by the Ethics Committee of our institution (Milano 2 approval n. 347/2010).

### 2.2 Body composition and nutritional status

Anthropometric measures included: body weight, height, body mass index (BMI), waist circumference (WC), mid arm circumference (MAC), tricipital and bicipital skinfold thickness (TST, BST; measured with a Harpenden skinfold caliper). The mid-arm muscle circumference (MAMC) was calculated as follows: MAMC (cm) = MAC (cm) − (πxTST (cm)); these measurements were taken on the dominant arm as described elsewhere (9).

Nutritional status was assessed using the Malnutrition Inflammation Score (MIS) and Protein Energy Wastage (PEW) criteria.

The MIS is an adaptation of the SGA questionnaire specifically for hemodialysis (HD) patients proposed by Amparo et al. (10) and Kalantar-Zadeh et al. (11). By adding some objective clinical and laboratory markers relevant to CKD, MIS transforms SGA into a semiquantitative scoring system. MIS has been validated against other nutritional/inflammatory biomarkers and is associated with poorer prognosis in patients on HD (10–12), peritoneal dialysis (13, 14), kidney transplantation (15) and in non-dialyzed CKD patients (10). The MIS is a composite score made up of 10 components, each with four levels of severity: from 0 (normal) to 3 (severely abnormal). A total score of 4–7 indicates risk of malnutrition and a score of ≥8 indicates malnutrition (16).

The diagnosis of PEW was made using the ISRNM criteria, which are divided into 4 distinct domains: serum chemistry, body mass, muscle mass, and dietary intake, with different indicators for each domain. A positive indicator in at least 3 of the domains is sufficient for the diagnosis of PEW (17).

Patients were divided into sLPI and NPI groups based on nPCR values (respectively: nPCR ≤0.8 g/kg or > 8 g/kg), which was estimated using the Maroni and Mitch formula (18).

Caloric intake was estimated using 3-day food diaries (filled in during the 3 days prior to the visits -Sunday to Tuesday) and then calculated using the Winfood nutritional software (Medimatica Srl, Teramo, Italy).

### 2.3 Frailty assessment

The frailty phenotype (FP) proposed by Fried et al. (19) was used for the assessment of frailty. Five components were used to define frailty: (1) involuntary weight loss ≥4.5 kg in 12 months; (2) exhaustion as feeling fatigued ≥4 days per week for more than 3 months; (3) weakness as handgrip strength <16 kg in women and < 27 kg in men; (4) slow walking as a 4-meter walk test speed >0.8 m/s; reduced physical activity as a score < 7 on a physical activity scale described elsewhere (11, 20). Patients with a score of three or more of the impaired items were classified as frail.

### 2.4 Physical performance

The Short Physical Performance Battery (SPPB) and handgrip strength were used to assess physical performance.

The SPPB includes: standing balance test, walking 4 meters, and time to get up from a chair five times (21). Each SPPB component test is scored from 0 to 4, with higher scores indicating better physical performance (22). Hand grip strength was measured using the Jamar dynamometer. Values <16 kg in women and < 27 kg in men were considered impaired (23).

### 2.5 Biochemical parameters

On the same days of the visits, all biochemical analyses to evaluate renal function, metabolic and nutritional status were performed in the central laboratory of our institution.

### 2.6 Detection of IL-6 serum levels, MCP-1 and TNF-alpha

Serum samples were frozen and stored at −80°C at the laboratory of nephrology of our Institution. Values were evaluated in duplicate by using enzyme-linked immunosorbent assay (ELISA) kits following the manufacturer’s instructions. We used these specific kits: Quantikine ELISA Human CCL2/MCP-1 Immunoassay DCP00, Human TNF-alpha ELISA Kit (Thermo Fisher Scientific, Monza, Italy), Quantikine HS ELISA Human IL-6 Immunoassay HS600B (R&D Systems, Space, Milano, Italy). Zero was included in each resulting curve as the last standard value. Results were validated by using Quantikine Immunoassay Control Group 1–4 or 10 (R&D Systems, Space, Milano, Italy). Absorbance readings were measured at 450 nm by spectrophotometer (Xenius Safas, Monaco).

### 2.7 Statistical analysis

All data are expressed as mean ± SD or median ± IQR, as appropriate. Between-group comparisons of parametric variables were made using Student’s *t*-test, while between-group comparisons of non-parametric variables were made using the Mann–Whitney U test. Intra-group comparisons of parametric variables were made using the paired t-test, while intra-group comparisons of non-parametric variables were made using the Wilcoxon test. Proportions and categorical variables were compared using the independent chi-squared test (χ^2^). Pearson or Spearman tests were used for regression analyses, as appropriate. Statistical significance was set at *p* < 0.05. SPSS software version 5.0.1 was used for all analyses.

## 3 Results

### 3.1 General population characteristics

We evaluated cross-sectionally 123 patients, whose general characteristics are depicted in Table 1.

**Table 1:** Comparison of general characteristics between patients with spontaneous low proteins intake and normal protein intake.

Variables	sLPI (*n* = 84)	NPI (*n* = 49)	*p*
Age, years	80 ± 8	75 ± 14	**0.017**
Males/Females, n (%)	60 (71)/24 (29)	36(73)/13 (27)	0.65
Weight, Kg	71 ± 13	76 ± 14	**0.048**
Diabetes, n (%)	29 (59)	36 (43)	0.075
Frailty, n (%)	28 (33)	12 (24)	**0.037**
eGFR, mL/min/1.73 m^2^	18[13–23]	28[22–38]	**<0.0001**
Creatinine Clearance, mL/min/1.73 m^2^	18[14–23.5]	39[23–46]	**<0.0001**

eGFR, estimated glomerular filtration rate. Data are expressed as mean ± standard deviation or median [interquartile range] when appropriate. Categorical variables are expressed as number, n, and percentages (%). *p*-values are intended for trend and values less than 0.05 are indicated in bold.

sLPI patients, were generally older (80 ± 8 vs. 75 ± 14, *p* = 0.017) and has a higher prevalence of frailty [24, (33%) vs. 12, (24%), *p* = 0.037]. These patients also had markedly worse renal function parameters, such as lower eGFR (18[13–23] vs. 28[22–38], *p* < 0.0001), creatinine clearance (18[14–23.5] vs. 39[23–46], *p* < 0.0001) and higher serum urea (107[83–132] vs. 80[67–119], *p* = 0.04). No differences in terms of sex and diabetes prevalence were observed.

### 3.2 Correlations of protein intake with nutritional status, and physical performance

First of all, patients with sLPI had lower BMI when compared to NPI patients (25.9[24.2–29.6] vs. 27.7[25.0–32.2], *p* = 0.038).

sLPI were significantly more malnourished compared to NPI when malnutrition was assessed both with MIS (25% in sLPI vs. 10%in NPI, *p* = 0.033) or PEW (43% in sLPI vs. 14% in NPI, *p* = 0.002). Moreover, nPCR levels were significantly lower in sLPI patients, when compared to NPI ones (0.63[0.51–0.69] vs. 0.95[0.87–1.1]; *p* < 0.0001, respectively) (Table 2).

**Table 2:** Correlations of protein intake with nutritional status, and physical performance.

Variables	sLPI (*n* = 84)	NPI (*n* = 49)	*p*
Nutritional characteristics			
PEW, n (%)	36(43)	7(14)	**0.002**
BMI, kg/m^2^	25.9[24.2–29.6]	27.7[25.0–32.2]	**0.038**
Malnutrition by MIS, n (%)	21(25)	5(10)	**0.033**
Urea, mg/dL	107[83–132]	80[67–119]	**0.04**
Total cholesterol, mg/dL	157[137–181]	160[144–186]	0.33
HDL-cholesterol, mg/dL	50[42–62]	51[41–60]	0.92
Triglycerides, mg/dL	111[83–148]	117[91–166]	0.24
Iron, μg/dL	66[53–84]	75[59–84]	0.094
Transferrin, mg/dL	219[197–249]	229[205–248]	0.26
Ferritin, ng/mL	117[64–238]	117[64–196]	0.47
Hb, g/dL	11.6[10.8–12.8]	13.0[12.1–13.8]	**<0.0001**
Albumin, g/dL	3.9[3.6–4.2]	4.0[3.8–4.2]	**0.029**
Prealbumin, mg/dL	28[22–32]	29[25–31]	0.58
Urinary urea 24 h, g/24 h	13.1[11.0–15.6]	21.7[18.4–24.3]	**<0.0001**
nPCR, g/kg/day	0.63[0.51–0.69]	0.95[0.87–1.1]	**<0.0001**
Energy intake, Kcal/Kg	19.4 [14.9–25.2]	21.2 [14.7–26.3]	0.45
Body composition and physical performance			
Handgrip, kg	21 [16–28]	21[16–29]	0.78
Handgrip reduction, n (%)	51(61)	27(55)	0.52
SPPB	7 [5–9]	9[7–10]	**0.004**
Gait test time, s	7.22 + 2.7	6.08 + 2	**0.04**
Sarcopenia, n (%)	21(25)	10(20)	0.83
MAMC, cm	23.6[21.8–26.1]	25.0[22.9–27.5]	**0.049**
OH, L	1.8[0.8–2.8]	0.6[−0.1–1.4]	**<0.0001**
LTI, kg/m^2^	13.9[11.3–16.1]	14.0[11.4–15.5]	0.85
FTI, kg/m^2^	11.1[8.8–13.5]	13.7[9.2–17.7]	**0.049**

PEW, Protein energy wasting; BMI, Body mass index; MIS, malnutrition inflammation score; HDL, high density lipoprotein; Hb, Hemoglobin; nPCR, normalized protein catabolic rate; SPPB, short physical performance battery, MAMC, mid arm muscle circumference; OH, overhydration; LTI, lean tissue index; FTI, fat tissue index. Data are expressed as mean ± standard deviation or median [interquartile range] when appropriate. Categorical variables are expressed as number, n, and percentages (%). *p*-values are intended for trend and values less than 0.05 are indicated in bold.

Concerning other biochemical nutritional markers, albumin (3.9[3.6–4.2] g/dL vs. 4.0[3.8–4.2] g/dL, *p* = 0.029), hemoglobin (11.6[10.8–12.8] vs. 13.0[12.1–13.8], *p* < 0.0001) and urinary urea excretion (13.1[11.0–15.6] vs. 21.7[18.4–24.3], *p* < 0.0001) were significantly lower in sLPI patients, when compared to NPI ones. No other differences were observed in terms of the remaining markers of iron status and regarding lipid profile (Table 2).

Important differences were also noticeable in terms of muscular mass and body composition between sLPI and NPI patients. Indeed, sLPI patients had lower MAMC (23.6[21.8–26.1] vs. 25.0[22.9–27.5], *p* = 0.049) and FTI (11.1[8.8–13.5] vs. 13.7[9.2–17.7], *p* = 0.049) and were more overhydrated (1.8[0.8–2.8] vs. 0.6[−0.1–1.4], *p* < 0.0001) when compared to NPI patients. No differences in terms of sarcopenia or LTI were noticed.

We also wanted to look for eventual differences in terms of physical performance among the patients of our cohort. sLPI had worse scores for physical performance compared to NPI patients evaluated as SPPB (7[5–9] vs. 9[7–10], *p* = 0.004) and gait test time (7.22 ± 2.7 vs. 6.08 ± 2, *p* = 0.04). Handgrip strength was not different among the two patients’ subgroups (Table 2).

A worse overall nutritional status was correlated with impaired physical activity parameters. In particular, a higher MIS score strongly correlated with a worse overall physical performance, represented by lower SPPB (Rho −0.36, *p* < 0.0001), handgrip strength (Rho −0.16, *p* = 0.049) and longer gait speed test (Rho 0.23, *p* = 0.012). Regarding the other main indicators of nutritional status, lower nPCR significantly correlated with lower SPPB (Rho 0.26, *p* = 0.006) but not with other physical performance parameters, while PEW seemed not to be correlated with patients’ performance (Table 3).

**Table 3:** Correlations of sLPI and MIS, PEW, nPCR with physical performance test.

Dependent variables		Variables	Rho	*p*
nPCR		SPPB	0.26	**0.006**
		Handgrip strength test	0.016	0.85
		Gait speed test	−0.09	0.31
MIS		SPPB	−0.36	**0.012**
		Handgrip strength test	−0.16	**0.049**
		Gait speed test	0.23	**0.012**
Dependent variables		Variables	Median [IQR]	*p*
PEW	Yes	SPPB	8.0 [5.5–9.5]	0.62
	No		8.0 [5.0–10.0]	
	Yes	Handgrip strength test	21.2 ± 6.7	0.40
	No		22.4 ± 8.3	
	Yes	Gait speed test	6.9 ± 2.7	0.71
	No		6.7 ± 2.4	

sLPI, low protein intake; MIS, malnutrition inflammation score; nPCR, normalized protein catabolic rate; PEW, Protein energy wasting; SPPB, short physical performance battery. Correlation analysis was performed using the Spearman (Rho) model for nPCR and MIS, and Kruskal-Wallis and ANOVA for association with PEW. *p*-values are intended for trend and values less than 0.05 are indicated in bold.

### 3.3 Correlations of protein intake with overall inflammatory status

Among inflammatory markers IL-6 was markedly higher in sLPI patients (4.6[2.9–8.9] vs. 2.8[0.8–5.1], *p* = 0.002) while other markers were not different between the two groups (Table 4).

**Table 4:** Comparison of inflammatory markers between the two study groups.

Variables	sLPI (*n* = 84)	NPI (*n* = 49)	*p*
CRP, mg/dL	0.25 [0.09–0.5]	0.23 [0.12–0.51]	0.78
IL-6, pg./mL	4.6 [2.9–8.9]	2.8 [0.8–5.1]	**0.002**
TNF-alpha, pg./mL	13.7 [9.5–19.4]	10.7 [7.8–16.1]	0.19
MCP-1, pg./mL	408.2 [308.9–520.2]	416.9 [285.7–558.8]	0.90

CRP, c-reactive protein; TNFα, Tumor necrosis factor alpha; MCP-1, Monocyte chemoattractant protein-1; IL-6, interleukin 6. Data are expressed as median [interquartile range]. *P* values are intended for trend and values less than 0.05 are indicated in bold.

When we evaluated the correlation between inflammatory markers and nPCR (Table 5), we found an inverse correlation of protein intake (nPCR) with both IL-6 and TNF-alpha (Rho −0.212, *p* = 0.022 and Rho −0.222, *p* = 0.034 respectively). We also evaluated the correlation between inflammatory markers and other markers of nutritional status. Higher IL-6 and lower MCP-1 were associated with PEW (5.1[2.7–10.7] vs. 4.1[2.2–6.3], *p* = 0.045 and 328.6[289.4–445.4] vs. 439.9[309.7–558.8], *p* = 0.049 respectively), while we did not find any correlation with MIS.

**Table 5:** Relationship between nutritional status markers and pro-inflammatory cytokines.

Dependent variables		Variables	Rho	*p*
nPCR		CRP	0.013	0.88
		IL-6	−0.212	**0.022**
		TNF-alpha	−0.222	**0.034**
		MCP-1	0.007	0.95
MIS		CRP	−0.012	0.89
		IL-6	−0.09	0.30
		TNF-alpha	−0.06	0.59
		MCP-1	−0.15	0.20
		Variables	Median [IQR]	*p*
PEW	Yes	CRP	0.31 [0.12–0.76]	0.17
	No		0.24 [0.09–0.48]	
	Yes	IL-6	5.1 [2.7–10.7]	**0.045**
	No		4.1 [2.2–6.3]	
	Yes	TNF-alpha	13.2 [9.5–20.5]	0.77
	No		13.6 [9.3–17.6]	
	Yes	MCP-1	328.6 [289.4–445.4]	**0.049**	No	439.9 [309.7–558.8]

PEW, Protein energy wasting; MIS, malnutrition inflammation score; nPCR, normalized protein catabolic rate; CRP, c-reactive protein; TNFα, Tumor necrosis factor alpha; MCP-1, Monocyte chemoattractant protein-1; IL-6, interleukin 6; IL-10, interleukin 10. Correlation analysis was performed using the Spearman (Rho) model for nPCR and MIS, and Kruskal-Wallis for association with PEW. *p*-values are intended for trend and values less than 0.05 are indicated in bold.

We also performed correlation between inflammatory markers and physical performance tests, but we did not find any correlation (Table 6).

**Table 6:** Correlations between inflammatory markers and the tests of physical performance.

Dependent variables	Variables	Rho	*p*
IL-6	SPPB	−0.012	0.90
	Handgrip strength test	0.11	0.23
	Gait speed test	0.020	0.84
TNF-alpha	SPPB	−0.082	0.47
	Handgrip strength test	−0.14	0.21
	Gait speed test	0.054	0.64
	SPPB	−0.032	0.78
MCP-1	Handgrip strength test	0.001	0.99
	Gait speed test	−0.049	0.68
CRP	SPPB	−0.085	0.34
	Handgrip strength test	0.005	0.96
	Gait speed test	0.088	0.34

CRP, c-reactive protein; TNFα, Tumor necrosis factor alpha; MCP-1, Monocyte chemoattractant protein-1; IL-6, interleukin 6; SPPB, short physical performance battery. Correlation analyses was performed using Spearman (Rho) model. *p*-values are intended for trend and values less than 0.05 are indicated in bold.

#### 3.3.1 sLPI is associated with malnutrition and worse physical performance

To confirm our findings, we performed a logistic regression evaluating the association between sLPI, malnutrition evaluated by MIS, frailty and physical performance parameters. Among the main risk factors for sLPI development, we inserted in this model eGFR and patients’ age, to correct for main confounders and to better understand the strength of eventual associations. sLPI was strongly associated with lower eGFR [OR, 0.9 (0.86–0.95), *p* < 0.0001]. However, sLPI remained associated with lower SPPB score [OR, 0.79 (0.46–0.97), *p* = 0.046] and malnutrition [1.6 (1.05–3.5), *p* = 0.041] independently from patients age and eGFR. The correlation between sLPI, frailty and handgrip was not confirmed in this analysis (Table 7).

**Table 7:** Logistic regression analysis evaluating the associations of sLPI with MIS and eGFR and physical performance tests.

Dependent variables	Variables	OR	*p*
sLPI	eGFR (mL/min/1.73 m^2^) Age (years) SPPB	0.9 (0.86–0.95) 1.02 (0.94–1.10) 0.79 (0.46–0.97)	**<0.0001** 0.61 **0.046**
	Handgrip strength test	1.05 (0.97–1.14)	0.76
	Malnutrition (MIS)	1.6 (1.05–3.5)	**0.041**
	Frailty	2.8 (0.52–15.2)	0.225

sLPI, low protein intake; eGFR, estimated glomerular filtration rate; MIS, malnutrition inflammation score; SPPB, short physical performance battery. Odds ratio (OR) are expressed as OR (95% confidence interval). *P* < 0.05 are in bold.

## 4 Discussion

Our study demonstrates that among elderly patients incident in an outpatients CKD clinic, almost two thirds have a spontaneous reduction in dietary protein intake. sLPI was also independently associated with malnutrition and worse performance status. In addition, sLPI was associated with systemic inflammation. Our findings support those of some other recent studies, which have highlighted the importance of personalizing the nutritional approach for elderly patients with CKD (24–29). The K-DOQI 2020 guidelines for nutrition in CKD recommend moderate to severe protein restriction from CKD stage 3 (1). Nutritional therapy based on reducing protein intake, together with a qualitative approach to nutrient selection, is currently the mainstay of nutritional management in CKD patients of all ages (1, 30). However, CKD is “*per se*” a major determinant of protein and energy wasting and it may synergistically act with other age-related risk factors and with the concurrent comorbidities to induce malnutrition in the elderly. A condition that is exacerbated when low protein consumption is accompanied by an inadvertent reduction in energy intake (31, 32). Therefore, these peculiarities may lead to a kind of trade-off in the elderly between the need to delay the progression of renal disease and the risk of malnutrition. In particular, the risk of malnutrition may become significant if the initial nutritional status and protein-energy requirements are not accurately assessed and adequate follow-up is not planned (33–36). However, despite these necessary cautions, the indication to reduce protein intake in patients with CKD is supported by clear pathophysiological considerations and reliable clinical evidence (30, 37–39).

The primary aim of our study was to investigate the clinical relevance of sLPI as a potential risk factor or surrogate for malnutrition. Strikingly, we documented that up to two-thirds of elderly patients with advanced CKD have sLPI and that this condition is associated with malnutrition, inflammation and reduced overall performance status. Therefore, the main concern arising from our findings is what dietary regimen should be used in elderly patients with sLPI. Previously, we showed in a pilot study that in elderly CKD patients at risk of malnutrition, prescribing a low-protein diet, if supported by adequate energy intake, is safe and may even lead to an overall improvement in nutritional status. However, in the current study, 25% of patients with sLPI were malnourished at MIS and 43% were affected by PEW, conditions in which LPD is contraindicated and indeed oral supplementation should be considered (1). Our data support the importance of carefully assessing the nutritional status of elderly CKD patients before prescribing a low protein diet, as recommended by the latest K-DIGO guidelines (1). However, we also recognize that a thorough nutritional assessment is time consuming and may be difficult to administer on a large scale. We propose the assessment of spontaneous protein intake derived from steady-state nPCR as a reliable and cost-effective marker to identify patients with sLPI, a condition that should prompt a more comprehensive nutritional assessment. As sLPI is often associated with malnutrition and PEW in elderly CKD patients, it may help to screen for patients who may benefit more from oral supplements rather than LPD (40–44). In these circumstances, supplementation with alpha-keto analogs and amino acid blends may also be a reasonable option for patients at risk or already affected by protein malnutrition (36, 40, 45).

Moreover, with regard to inflammatory markers, although CRP is the most commonly used marker in clinical practice due to its limited cost and ease of detection, part of the message of our article includes that other inflammatory markers could also be routinely included for the assessment of the nutritional and metabolic status of the CKD patient. Indeed, among patients with CKD, the coexistence of malnutrition and inflammation is a well-recognized condition (46–50).

Overall, our data support the evidence that the prescription of appropriate nutritional interventions in elderly patients with CKD should follow a stepwise approach based on individual assessment of renal inflammatory and nutritional status. Therefore, different combinations of renal and nutritional parameters may configure different scenarios of relative risks and benefits, and clinical decisions should prioritize renal or nutritional issues as needed. We are aware that there are several limitations to our study. Firstly, the associations of sLPI with malnutrition, inflammation and exercise capacity in elderly CKD patients cannot be attributed to causality because of the cross-sectional design. Second, our study is monocentric and our population is relatively small. However, by using a highly standardized protocol for patient selection, biochemical analyses and clinical observations, the monocentric nature of our study allowed us to reduce potential sources of bias. In particular, we excluded patients who might have spontaneously reduced their protein intake due to certain clinical conditions by using strict inclusion and exclusion criteria. To our knowledge, this is the first study to comprehensively assess the relationship among SLPI, nutritional status, inflammation, Physical performance and frailty in older non-dialyzed patients with advanced CKD.

## 5 Conclusion

In conclusion, we found that in older patients with advanced CKD, up to 68% had low spontaneous protein intake and were frailer, malnourished and had worse overall physical performance. These findings emphasize the importance of assessing patients’ needs, and personalized approaches with individual risk–benefit assessments should be sought. nPCR and MIS are simple and easy tools to assess protein intake and malnutrition that could be used by those who are not able to perform a refined assessment of patients’ nutritional status and protein and calorie intakes, and could ensure a sound nutritional approach in daily clinical practice for a large population of elderly CKD patients.

## Data availability statement

The datasets analyzed for this study can be found in the OSF repository (https://osf.io/m8uyd/?view_only=45bae23631794e24a855ff3cebaccffc).

## Ethics statement

The studies involving humans were approved by the Ethics Committee of our Institution (Milano 2- approval no. 347/2010). The studies were conducted in accordance with the local legislation and institutional requirements. Written informed consent for participation in this study was provided by the participants’ legal guardians/next of kin. Written informed consent was obtained from the individuals for the publication of any potentially identifiable images or data included in this article.

## Author contributions

SV: Conceptualization, Investigation, Project administration, Validation, Visualization, Writing – original draft, Writing – review & editing. PM: Conceptualization, Data curation, Formal analysis, Methodology, Software, Writing – original draft. SA: Methodology, Writing – original draft. GC: Funding acquisition, Resources, Writing – review & editing. LC: Data curation, Formal analysis, Investigation, Methodology, Software, Writing – original draft.
